# Supplementary material for: Morphometric and molecular discrimination of the sugarcane aphid, Melanaphis sacchari, (Zehntner, 1897) and the sorghum aphid Melanaphis sorghi (Theobald, 1904)
Source: PLoS One. 2021 Mar 25;16(3):e0241881. doi: 10.1371/journal.pone.0241881 (PMC7993840; doi:10.1371/journal.pone.0241881)
Supplement: S1 Fig — (PDF) [file pone.0241881.s001.pdf]

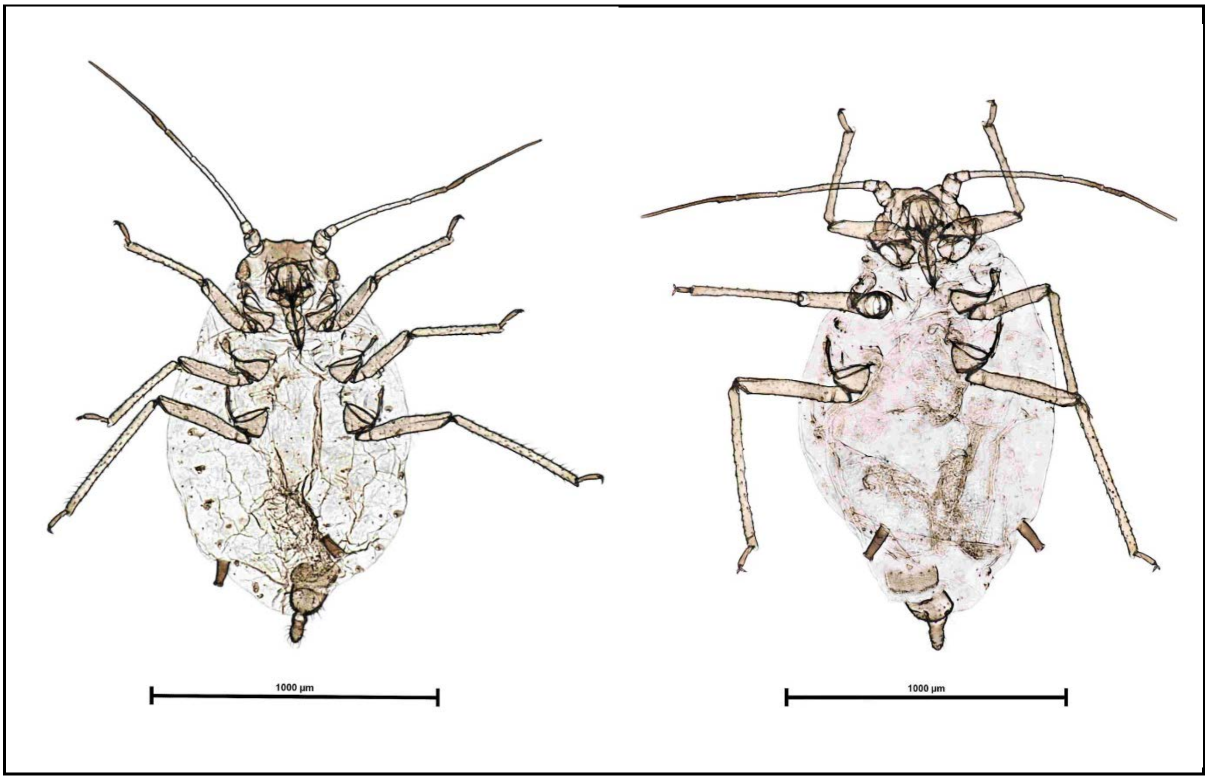

S1 Fig. *Melanaphis* apterous female habitus. Left: *Melanaphis sacchari* specimen SNIB00025_0101 from Ecuador (*HindTibia:pt* = 1.63; *pt:siph* = 3.52; *pt:cauda* = 2.46). Right *Melanaphis sorghi* specimen SNIB00004_0101 from Benin (*HindTibia:pt* = 2.08; *pt:siph* = 2.26; *pt:cauda* = 1.94). The pictures were cleaned and optimized (removal of spots and debris from the background) using the Adobe Photoshop software.
