# Supplementary figures and images for: Morphometric and molecular discrimination of the sugarcane aphid, Melanaphis sacchari, (Zehntner, 1897) and the sorghum aphid Melanaphis sorghi (Theobald, 1904)
Source: PLoS One. 2021 Mar 25;16(3):e0241881. doi: 10.1371/journal.pone.0241881 (PMC7993840; doi:10.1371/journal.pone.0241881)

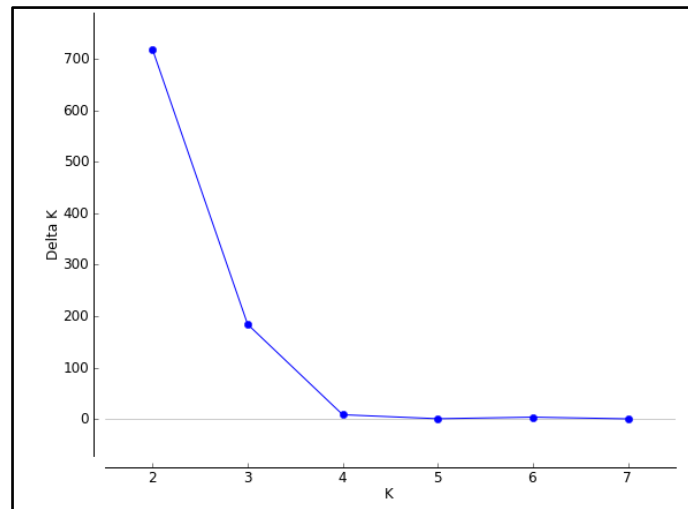

S2 Fig. Evanno method inferring  $k = 2$ .

Supplement: S2 Fig — (PDF) [file pone.0241881.s002.pdf]
