# Supplementary material for: Morphometric and molecular discrimination of the sugarcane aphid, Melanaphis sacchari, (Zehntner, 1897) and the sorghum aphid Melanaphis sorghi (Theobald, 1904)
Source: PLoS One. 2021 Mar 25;16(3):e0241881. doi: 10.1371/journal.pone.0241881 (PMC7993840; doi:10.1371/journal.pone.0241881)
Supplement: S1 Raw image — (PDF) [file pone.0241881.s003.pdf]

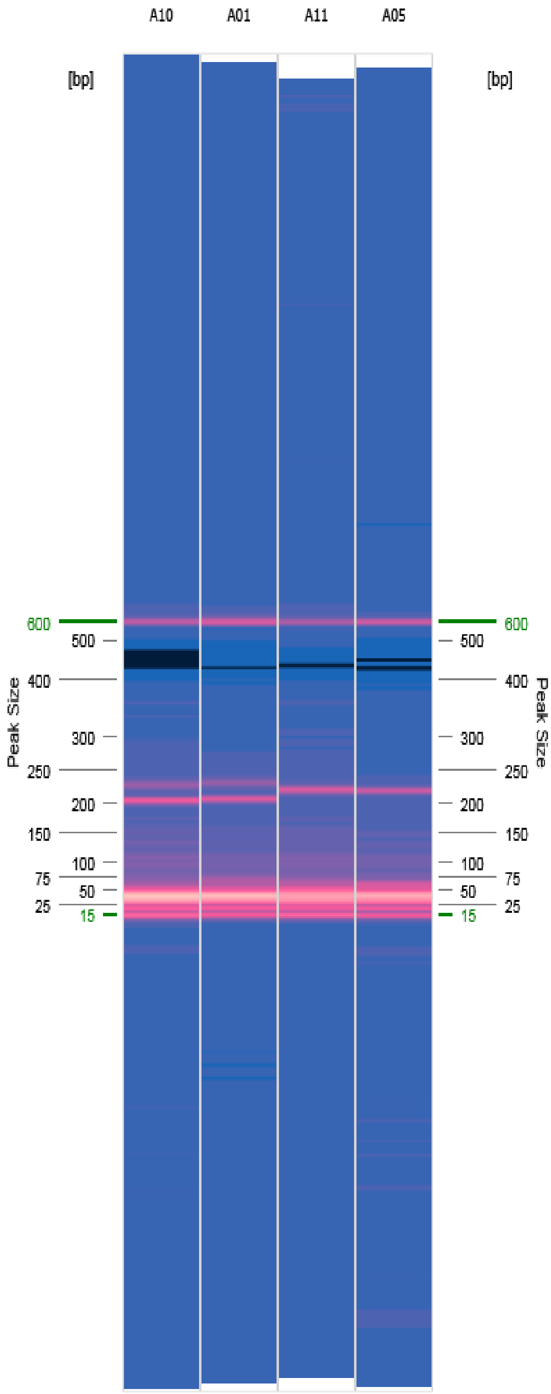

S1 Raw image. Original image used to draw Fig 8. Molecular diagnosis for separation of *M. sacchari* and *M. sorghi* using the SSR locus CIR-Ms-G01.
M. sacchari (MLL-D) are in lanes A10 (voucher # SNIB00040_0101) and A1 (voucher # SNIB00233_0102). *M. sorghi* (MLL-F) are in lanes A11 (voucher # SNIB00075_0101) and A5 (voucher # SNIB000237_0102).
The image was generated by the Qiaxcel ScreenGel 1.6.0 software
