# Supplementary material for: Morphometric and molecular discrimination of the sugarcane aphid, Melanaphis sacchari, (Zehntner, 1897) and the sorghum aphid Melanaphis sorghi (Theobald, 1904)
Source: PLoS One. 2021 Mar 25;16(3):e0241881. doi: 10.1371/journal.pone.0241881 (PMC7993840; doi:10.1371/journal.pone.0241881)
Supplement: S2 Table — (PDF) [file pone.0241881.s005.pdf]

S2 Table. Comparison of morphological characteristics of *M. sacchari* and *M. sorghi* viviparous alate females. Measures are in μm or unitless. n = maximum number of specimens observed, the actual number depends on the missing data within each trait.

	M. sacchari n = 5 (3 samples)	*M. sorghi* n = 5 (3 samples)
BL	1450 (1200-1638)	1550 (1463-1713)
cauda	108 (94-113)	125 (106-138)
caudaBW	84 (75-94)	100 (88-113)
urs	75 (75-75)	78 (75-81)
NsetaeCauda	8.8 (7-10)	9.2 (8-10)
htII	74 (69-81)	75 (72-81)
HindFemur	337 (275-406)	356 (338-413)
HindTibia	626 (531-738)	689 (656-775)
HindTibiaW	27 (25-31)	28 (25-35)
siph	84 (69-97)	105 (97-113)
siphDW	33 (30-38)	35 (31-40)
siphBW	36 (25-44)	39 (28-56)
AntI	56 (53-63)	56 (50-59)
AntII	46 (41-53)	50 (45-53)
AntIII_IV	422 (347-522)	391 (344-450)
AntV	188 (153-238)	168 (150-181)
VIb	93 (84-100)	86 (81-90)
pt	316 (291-375)	305 (300-325)
AntIIIBW	25 (19-31)	28 (25-30)
NsetaeAntIII_IV	2.5 (2-5)	3.0 (1-6)
NsetaeAntV	1.6 (1-3)	1.9 (1-3)
NRhinAntIII	10.5 (8-14)	6.8 (6-10)
NRhinAntIV	0.0 (0-0)	0.0 (0-0)
NRhinAntV	1.0 (1-1)	1.0 (1-1)
Ant	1121 (977-1350)	1045 (975-1147)
Ant:BL	0.77 (0.67-0.82)	0.67 (0.65-0.7)
urs:htII	1.01 (0.92-1.09)	1.03 (0.92-1.08)
pt:VIb	3.42 (2.91-3.75)	3.57 (3.33-3.71)
pt:cauda	2.93 (2.58-3.33)	2.45 (2.29-2.82)
pt:siph	3.78 (3.05-4.32)	2.92 (2.7-3.1)
HindTibia:pt	1.99 (1.79-2.34)	2.26 (2.19-2.38)
cauda:urs	1.44 (1.25-1.5)	1.62 (1.31-1.75)
urs:VIb	0.82 (0.75-0.89)	0.91 (0.83-1)
siph:BL	0.06 (0.05-0.06)	0.07 (0.06-0.08)
siph:siphBW	2.49 (1.71-3.88)	2.84 (2.00-3.56)
siph:cauda	0.78 (0.69-0.86)	0.84 (0.74-0.94)
cauda:caudaBW	1.29 (1.2-1.38)	1.25 (1.13-1.43)
