# Supplementary material for: Morphometric and molecular discrimination of the sugarcane aphid, Melanaphis sacchari, (Zehntner, 1897) and the sorghum aphid Melanaphis sorghi (Theobald, 1904)
Source: PLoS One. 2021 Mar 25;16(3):e0241881. doi: 10.1371/journal.pone.0241881 (PMC7993840; doi:10.1371/journal.pone.0241881)
Supplement: S3 Table — (PDF) [file pone.0241881.s006.pdf]

S3 Table. CO1 haplotypes, position and nature of nucleotide substitutions.

Haplotype	position (bp)			
	263	294	343	531
H1	C	G	A	C
H2	T	G	G	C
H3	C	A	G	C
H6	C	G	A	A
