# Supplementary material for: Morphometric and molecular discrimination of the sugarcane aphid, Melanaphis sacchari, (Zehntner, 1897) and the sorghum aphid Melanaphis sorghi (Theobald, 1904)
Source: PLoS One. 2021 Mar 25;16(3):e0241881. doi: 10.1371/journal.pone.0241881 (PMC7993840; doi:10.1371/journal.pone.0241881)
Supplement: S4 Table — (PDF) [file pone.0241881.s007.pdf]

S4 Table. CO2 haplotypes, position and nature of nucleotide substitutions.

Haplotype	position (bp)			
	193	226	382	676
H1	A	T	T	T
H2	G	T	T	C
H3	A	T	T	C
H4	A	T	C	C
H5	A	C	T	C
