# Supplementary material for: Morphometric and molecular discrimination of the sugarcane aphid, Melanaphis sacchari, (Zehntner, 1897) and the sorghum aphid Melanaphis sorghi (Theobald, 1904)
Source: PLoS One. 2021 Mar 25;16(3):e0241881. doi: 10.1371/journal.pone.0241881 (PMC7993840; doi:10.1371/journal.pone.0241881)
Supplement: S5 Table — (PDF) [file pone.0241881.s008.pdf]

S5 Table. EF1-α haplotypes, position and nature of nucleotide substitutions. Ambiguous positions were heterozygous loci occur are signaled by *. Haplotypes bearing the same number (i.e. Hn) are identical when omitting the ambiguous positions.

| Haplotype | position (bp) | | | | |
|---|---|---|---|---|---|
| | 296* | 478* | 637 | 663* | 735* |
| H1a (n = 3) | T | A | T | A | C |
| H1c (n = 80) | T | A | T | R | C |
| H2b (n = 1) | T | T | A | G | C |
| H2a (n = 1) | T | A | A | G | T |
| H2c (n = 12) | Y | T | A | G | C |
| H2d (n = 13) | T | W | A | G | C |
| H2e (n = 61) | T | W | A | G | Y |
