# Supplementary material for: Morphometric and molecular discrimination of the sugarcane aphid, Melanaphis sacchari, (Zehntner, 1897) and the sorghum aphid Melanaphis sorghi (Theobald, 1904)
Source: PLoS One. 2021 Mar 25;16(3):e0241881. doi: 10.1371/journal.pone.0241881 (PMC7993840; doi:10.1371/journal.pone.0241881)
Supplement: S6 Table — (PDF) [file pone.0241881.s009.pdf]

S6 Table. Observed microsatellite Multi Locus Genotypes (MLG): allele size (bp) at each locus, distribution in each of the six Multi Locus Lineages (MLL). Species assignment of MLL-B and MLL-E requires confirmation.

Species	MLL	MLG	Locus								
			**CIR-Ms-G08**	**CIR-Ms-G403**	**CIR-Ms-G02**	**CIR-Ms-C08**	**CIR-Ms-G01**	**CIR-Ms-E01**	**CIR-Ms-G12**	**CIR-Ms-E03**	**CIR-Ms-D02**
M. sorghi	**A**	Ms30	229 / 229	253 / 253	250 / 318	189 / 203	204 / 206	245 / 247	214 / 216	176 / 176	220 / 228
		Ms301	229 / 229	253 / 253	248 / 312	189 / 203	206 / 206	245 / 247	214 / 216	176 / 176	220 / 228
		Ms302	229 / 229	253 / 253	248 / 314	189 / 203	206 / 206	245 / 247	214 / 216	176 / 176	220 / 228
		Ms303	229 / 229	253 / 253	250 / 298	189 / 203	206 / 206	245 / 247	214 / 216	176 / 176	220 / 228
		Ms304	229 / 229	253 / 253	250 / 310	189 / 203	206 / 206	245 / 247	214 / 216	176 / 176	220 / 228
		Ms305	229 / 229	253 / 253	250 / 312	189 / 203	206 / 206	245 / 247	214 / 216	176 / 176	220 / 228
		Ms306	229 / 229	253 / 253	250 / 314	189 / 203	206 / 206	245 / 247	214 / 216	176 / 176	220 / 228
		Ms307	229 / 229	253 / 253	250 / 316	189 / 203	206 / 206	245 / 247	214 / 216	176 / 176	220 / 228
		Ms308	229 / 229	253 / 253	250 / 318	189 / 203	206 / 206	245 / 247	214 / 216	176 / 176	220 / 228
		Ms309	229 / 229	253 / 253	250 / 343	189 / 203	206 / 206	245 / 247	214 / 216	176 / 176	220 / 228
		Ms31	229 / 229	253 / 253	254 / 314	189 / 203	206 / 206	245 / 247	214 / 216	176 / 176	228 / 230
		Ms310	229 / 229	253 / 253	250 / 345	189 / 203	206 / 206	245 / 247	214 / 216	176 / 176	220 / 228
		Ms311	229 / 229	253 / 253	252 / 312	189 / 203	206 / 206	245 / 247	214 / 216	176 / 176	220 / 228
		Ms312	229 / 229	253 / 253	252 / 314	189 / 203	206 / 206	245 / 247	214 / 216	176 / 176	220 / 228
		Ms313	229 / 229	253 / 253	254 / 312	189 / 203	206 / 206	245 / 247	214 / 216	176 / 176	220 / 228
		Ms314	229 / 229	253 / 253	254 / 314	189 / 203	206 / 206	245 / 247	214 / 216	176 / 176	220 / 228
		Ms315	229 / 229	253 / 253	256 / 314	189 / 203	206 / 206	245 / 247	214 / 216	176 / 176	220 / 228
		Ms316	229 / 229	253 / 253	246 / 312	189 / 203	206 / 206	245 / 247	214 / 216	176 / 176	220 / 228
		Ms317	229 / 229	253 / 253	246 / 316	189 / 203	206 / 206	245 / 247	214 / 216	176 / 176	220 / 228
		Ms41	229 / 229	253 / 253	252 / 314	189 / 203	206 / 206	245 / 247	214 / 216	176 / 176	220 / 226
		Ms42	229 / 229	253 / 253	254 / 314	189 / 203	206 / 206	245 / 247	214 / 216	176 / 176	220 / 226
		Ms5	229 / 229	253 / 253	250 / 316	189 / 203	206 / 210	245 / 247	214 / 216	176 / 176	220 / 228
	**E**	Ms32	229 / 229	253 / 253	216 / 216	197 / 197	198 / 206	245 / 245	208 / 212	216 / 224	220 / 222
		Ms33	229 / 229	253 / 253	216 / 216	197 / 197	198 / 206	245 / 245	208 / 212	224 / 224	220 / 222
	**F**	Ms50	227 / 229	253 / 253	199 / 199	199 / 203	198 / 198	245 / 245	212 / 216	174 / 174	220 / 222
		Ms51	227 / 227	253 / 253	199 / 199	199 / 203	198 / 198	245 / 245	212 / 216	174 / 174	220 / 222
		Ms52	227 / 229	251 / 253	199 / 199	199 / 203	198 / 198	245 / 245	212 / 216	174 / 174	220 / 222
		Ms53	227 / 227	255 / 255	199 / 199	199 / 203	198 / 198	245 / 245	212 / 216	174 / 174	220 / 222
		Ms54	227 / 229	255 / 255	199 / 199	199 / 203	198 / 198	245 / 245	212 / 216	174 / 174	220 / 222
		Ms55	227 / 229	253 / 253	199 / 199	199 / 203	198 / 198	245 / 245	216 / 216	174 / 174	220 / 222
		Ms56	227 / 227	255 / 255	199 / 199	203 / 203	198 / 198	245 / 245	212 / 216	174 / 174	220 / 222
		Ms57	227 / 227	253 / 253	199 / 199	197 / 201	198 / 198	245 / 245	212 / 216	174 / 174	220 / 222
		Ms58	227 / 227	251 / 251	199 / 199	197 / 201	198 / 198	245 / 245	212 / 216	174 / 174	220 / 222

Species	MLL	MLG	Locus								
			**CIR-Ms-G08**	**CIR-Ms-G403**	**CIR-Ms-G02**	**CIR-Ms-C08**	**CIR-Ms-G01**	**CIR-Ms-E01**	**CIR-Ms-G12**	**CIR-Ms-E03**	**CIR-Ms-D02**
M. sacchari	**B**	Ms1	233 / 233	253 / 259	199 / 199	197 / 199	185 / 206	247 / 247	212 / 216	188 / 193	226 / 232
		Ms121	233 / 233	253 / 259	199 / 199	197 / 199	185 / 206	247 / 247	212 / 216	188 / 191	226 / 232
		Ms122	233 / 233	253 / 259	199 / 201	197 / 199	185 / 206	247 / 247	212 / 216	188 / 191	226 / 232
		Ms2	233 / 233	253 / 259	199 / 199	197 / 199	185 / 206	247 / 247	212 / 216	188 / 193	226 / 252
	**C**	Ms11	233 / 233	251 / 259	199 / 199	197 / 199	185 / 210	247 / 247	212 / 216	186 / 193	228 / 232
		Ms12	233 / 233	251 / 259	199 / 205	195 / 197	185 / 210	247 / 247	212 / 216	186 / 193	228 / 232
		Ms13	233 / 233	251 / 259	199 / 201	197 / 199	185 / 210	247 / 247	212 / 216	186 / 201	228 / 232
		Ms14	233 / 233	251 / 259	199 / 203	195 / 197	185 / 210	247 / 249	212 / 216	186 / 193	228 / 232
		Ms15	233 / 233	251 / 259	199 / 201	197 / 199	185 / 210	247 / 247	212 / 216	186 / 193	228 / 232
		Ms16	233 / 233	251 / 259	199 / 203	195 / 197	185 / 210	247 / 247	212 / 216	186 / 193	228 / 232
		Ms17	233 / 233	251 / 259	199 / 201	197 / 199	185 / 210	247 / 247	212 / 216	186 / 193	224 / 228
		Ms18	233 / 233	251 / 259	199 / 201	197 / 201	185 / 210	247 / 247	212 / 216	186 / 193	228 / 232
		Ms19	233 / 233	251 / 259	199 / 203	195 / 197	185 / 210	247 / 247	212 / 216	186 / 193	220 / 228
		Ms20	233 / 233	251 / 259	199 / 203	195 / 197	185 / 210	247 / 247	212 / 216	186 / 193	228 / 230
		Ms21	233 / 233	251 / 259	199 / 203	197 / 195	185 / 210	247 / 247	212 / 216	186 / 193	228 / 234
		Ms22	233 / 233	251 / 259	199 / 199	197 / 199	185 / 210	247 / 247	212 / 216	186 / 193	220 / 232
		Ms23	233 / 233	251 / 259	199 / 199	197 / 199	185 / 210	247 / 247	212 / 216	188 / 193	228 / 232
		Ms24	233 / 233	251 / 259	199 / 199	197 / 199	185 / 210	247 / 247	212 / 218	186 / 193	228 / 232
		Ms25	233 / 233	251 / 259	199 / 199	197 / 199	185 / 210	247 / 247	212 / 216	186 / 193	226 / 234
		Ms26	233 / 233	251 / 259	199 / 199	197 / 199	185 / 210	247 / 247	212 / 216	186 / 193	226 / 232
		Ms6	233 / 233	251 / 259	199 / 199	197 / 199	185 / 210	247 / 247	212 / 216	186 / 193	228 / 234
		Ms7	233 / 233	251 / 259	199 / 199	197 / 199	185 / 210	247 / 247	204 / 212	186 / 193	228 / 232
		Ms8	233 / 233	251 / 259	199 / 199	197 / 199	185 / 212	247 / 247	212 / 216	186 / 193	228 / 232
	**D**	Ms10	233 / 233	251 / 259	201 / 201	197 / 199	185 / 206	247 / 247	212 / 218	186 / 188	226 / 234
		Ms102	233 / 233	251 / 259	201 / 201	197 / 201	185 / 206	247 / 247	212 / 216	186 / 188	226 / 234
		Ms9	233 / 233	251 / 259	201 / 201	197 / 199	185 / 206	247 / 247	212 / 216	186 / 188	226 / 234
