# Supplementary material for: Morphometric and molecular discrimination of the sugarcane aphid, Melanaphis sacchari, (Zehntner, 1897) and the sorghum aphid Melanaphis sorghi (Theobald, 1904)
Source: PLoS One. 2021 Mar 25;16(3):e0241881. doi: 10.1371/journal.pone.0241881 (PMC7993840; doi:10.1371/journal.pone.0241881)
Supplement: Attachment — Submitted filename: Response to Reviewers.docx [file pone.0241881.s010.docx]

**Reviewer #1:** The presented manuscript is an excellent example of combine morphological/molecular study resolving the problem of identification of the two important pest species of aphids. The Material and Method section is fully and precisely described. The introduction and the discussion are adequate.

My only advice is to change the order in the Results section as should be started with the molecular ones as it was described in the M&M.

- We agree that M&M and Results should follow the same order. We have preferred to change the order of the M&M section to correspond to that of Results section (this modification is not in track change mode so as not to disturb readability).

In the Table S1 in the locality the United States should be added as now are listed countries and states.

- We have added these data in the S1 Table.

S2 Table should be included into the main manuscript as there are important data concerning identification of the studied species.

- We have moved S2 Table to the Results section, as Table 2 (this modification is not in track change mode so as not to disturb readability).

**Reviewer #2:** The discrimination study of sugarcane aphid was performed with great care and up to date methods and statistics. The manuscript is well written and can easily be understood. The observations are interesting and important for our community.
The results show the importance of using more than one marker or taxonomic indicator for species identification or discrimination, which in my personal opinion should be a standard procedure! I congratulate the authors for a well done study and recommend publication.

- No modification requested.
